# Supplementary figures and images for: Interactive visualization of whole eukaryote genome alignments using NCBI’s Comparative Genome Viewer (CGV)
Source: bioRxiv. 2023 Nov 29:2023.10.30.564672. Preprint. [Version 2] doi: 10.1101/2023.10.30.564672 (PMC10705539; doi:10.1101/2023.10.30.564672)

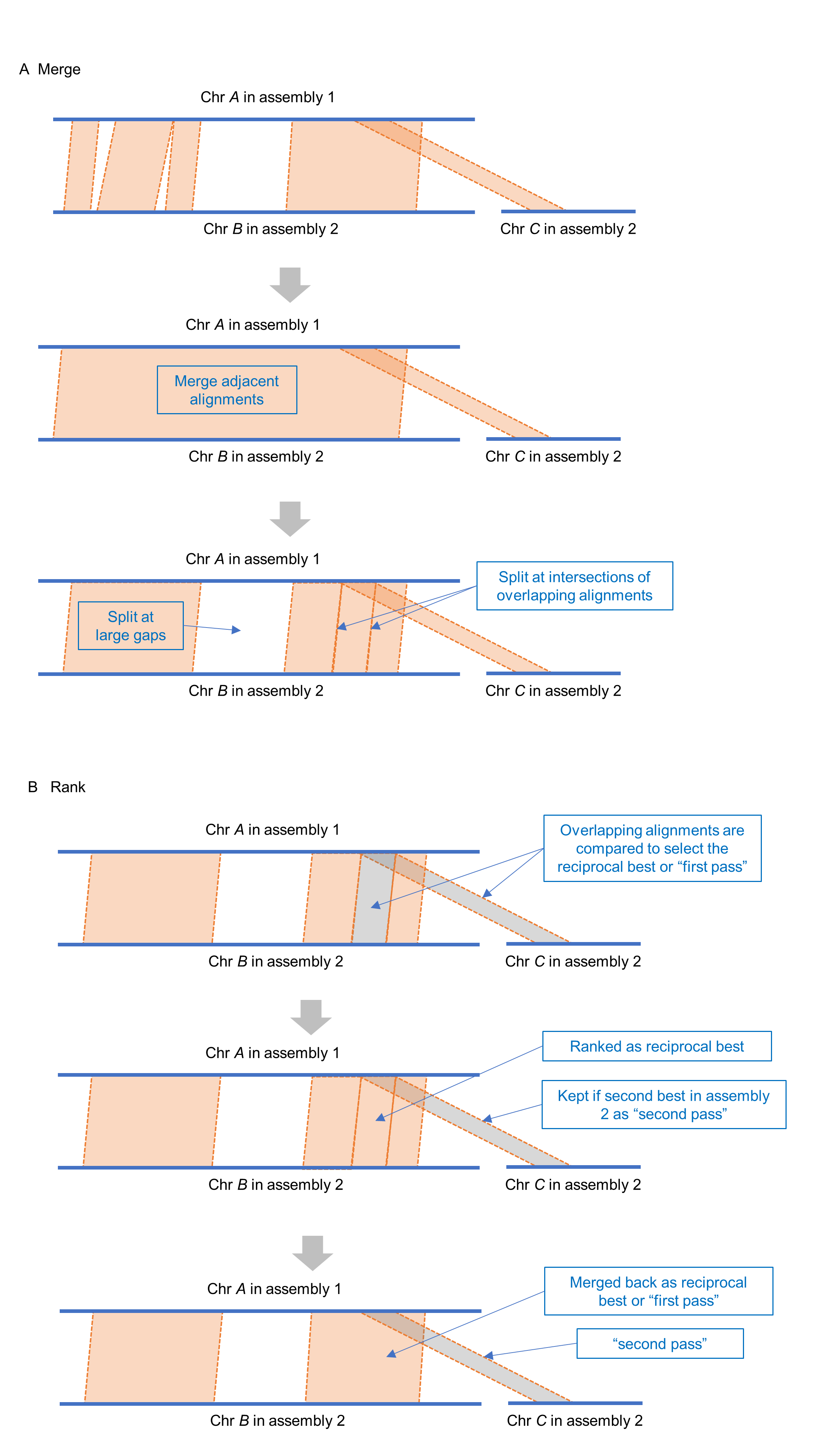

Supplement: Supplement 1 — S1 Figure. Merging, sorting, and ranking assembly-assembly alignments. (A) Flowchart showing that adjacent alignment segments are merged. Subsequently, alignments are split once again at large gaps. (B) Flowchart showing how overlapping alignments are separated, ranked, and re-merged. Reciprocal best-placed alignments are designated as “first pass”, while the non-best placed alignment is designated “second pass”. [file media-1.tif]
